# Association between right ventricular dysfunction and mortality in COVID‐19 patients: A systematic review and meta‐analysis

**DOI:** 10.1002/clc.23719

**Published:** 2021-09-16

**Authors:** Carlos Diaz‐Arocutipa, Jose Saucedo‐Chinchay, Edgar Argulian

**Affiliations:** ^1^ Vicerrectorado de Investigación Universidad San Ignacio de Loyola Lima Peru; ^2^ Asociación para el Desarrollo de la Investigación Estudiantil en Ciencias de la Salud (ADIECS) Lima Peru; ^3^ Programa de Atención Domiciliaria (PADOMI) Lima Peru; ^4^ Department of Emergency Hospital Nacional Edgardo Rebagliati Martins Lima Peru; ^5^ Mount Sinai Heart, Icahn School of Medicine at Mount Sinai New York USA

**Keywords:** coronavirus disease 2019, mortality, right ventricular dysfunction, systematic review

## Abstract

There is limited evidence about the prognostic utility of right ventricular dysfunction (RVD) in patients with coronavirus disease 2019 (COVID‐19). We assessed the association between RVD and mortality in COVID‐19 patients. We searched electronic databases from inception to February 15, 2021. RVD was defined based on the following echocardiographic variables: tricuspid annular plane systolic excursion (TAPSE), tricuspid S′ peak systolic velocity, fractional area change (FAC), and right ventricular free wall longitudinal strain (RVFWLS). All meta‐analyses were performed using a random‐effects model. Nineteen cohort studies involving 2307 patients were included. The mean age ranged from 59 to 72 years and 65% of patients were male. TAPSE (mean difference [MD], −3.13 mm; 95% confidence interval [CI], −4.08–−2.19), tricuspid S′ peak systolic velocity (MD, −0.88 cm/s; 95% CI, −1.68 to −0.08), FAC (MD, −3.47%; 95% CI, −6.21 to −0.72), and RVFWLS (MD, −5.83%; 95% CI, −7.47–−4.20) were significantly lower in nonsurvivors compared to survivors. Each 1 mm decrease in TAPSE (adjusted hazard ratio [aHR], 1.22; 95% CI, 1.08–1.37), 1% decrease in FAC (aHR, 1.09; 95% CI, 1.04–1.14), and 1% increase in RVFWLS (aHR, 1.33; 95% CI, 1.19–1.48) were independently associated with higher mortality. RVD was significantly associated with higher mortality using unadjusted risk ratio (2.05; 95% CI, 1.27–3.31), unadjusted hazard ratio (3.37; 95% CI, 1.72–6.62), and adjusted hazard ratio (aHR, 2.75; 95% CI, 1.52–4.96). Our study shows that echocardiographic parameters of RVD were associated with an increased risk of mortality in COVID‐19 patients.

## 1 INTRODUCTION

Coronavirus disease 2019 (COVID‐19), which is caused by severe acute respiratory syndrome coronavirus 2 (SARS‐CoV‐2), continues to be a public health problem across the globe.^1^ Although the lungs seem to be the main target of SARS‐CoV‐2 infection, there are a variety of cardiovascular presentations provoked directly or indirectly by SARS‐CoV‐2, including acute myocardial infarction, heart failure, arrhythmias, and myocarditis/pericarditis.^2^ The right ventricular compromise is increasingly recognized as an important complication in COVID‐19 patients.^3^ However, there is conflicted data about its prognostic utility in this population. Therefore, we performed a systematic review and meta‐analysis to assess the association between right ventricular dysfunction (RVD) and mortality in COVID‐19 patients.

## 2 METHODS

This review was reported according to the preferred reporting items for systematic reviews and meta‐analyses statement.^4^

### 2.1 Search strategy

We searched in the following databases: PubMed, Embase, Scopus, and Web of Science. The search was conducted from inception to February 15, 2021. The complete search strategy is available in Table S1. There were no restrictions on publication date or language. Furthermore, we performed a manual search of reference lists of included studies and relevant reviews to identify additional studies.

### 2.2 Eligibility criteria

The inclusion criteria were the following: (i) Cohort studies that included adult patients (≥18 years old) diagnosed with COVID‐19 by reverse transcription‐polymerase chain reaction and (ii) Studies that assessed the association between RVD assessed by echocardiography and mortality. We excluded commentaries, conference abstracts, systematic reviews, and narrative reviews.

### 2.3 Study selection

We downloaded all articles from electronic search to EndNote X8 software and duplicate records were removed. Titles and abstracts were independently screened by two review authors (Carlos Diaz‐Arocutipa and Jose Saucedo‐Chinchay) to identify relevant articles. Additionally, the same review authors (Carlos Diaz‐Arocutipa and Jose Saucedo‐Chinchay) independently examined the full‐text of each article and registered reasons for the exclusion. Any disagreement on title/abstract and full‐text selection was resolved by consensus.

### 2.4 Echocardiographic parameters

We assess any of the following echocardiographic parameters: tricuspid annular plane systolic excursion (TAPSE), tricuspid S′ peak systolic velocity, fractional area change (FAC), and right ventricular free wall longitudinal strain (RVFWLS). In addition, if available, the investigator‐defined RVD was also evaluated.

### 2.5 Data extraction

The information from each study was independently extracted by two review authors (Carlos Diaz‐Arocutipa and Jose Saucedo‐Chinchay) using a standardized data extraction form that was previously piloted. Any disagreement was resolved by consensus. We extracted the following data: first author name, year of publication, country, study design, sample size, population, age, sex, comorbidities, timing of echocardiographic evaluation, echocardiographic parameters of right ventricular function, and mortality.

### 2.6 Risk of bias assessment

The Newcastle‐Ottawa scale (NOS) was used to assess the risk of bias of cohort studies.^5^ Each study was classified into the following groups: low risk of bias (8–9 points), moderate risk of bias (5–7 points), and high risk of bias (0–4 points). The risk of bias was independently evaluated by two review authors (Carlos Diaz‐Arocutipa and Jose Saucedo‐Chinchay) and any disagreement was resolved by consensus.

### 2.7 Statistical analyses

We performed all meta‐analyses using a random‐effects model. The between‐study variance was estimated using the Paule‐Mandel estimator.^6^ We pooled unadjusted and adjusted effect measures for binary variables and mean differences (MD) with their 95% confidence interval (95% CI) for continuous variables. In the case of studies that have only reported median and interquartile range, the mean and SD were estimated using the method published by Wan et al.^7^ Heterogeneity among studies was evaluated using the chi‐squared test (threshold *p* < .10) and I^2^ statistic. Heterogeneity was defined as low if I^2^ < 30%, moderate if I^2^ = 30%–60%, and high if I^2^ > 60%. Publications bias was assessed by visual inspection of funnel plots. Statistical tests for funnel plot asymmetry were not performed since at least 10 studies per outcome are required. Subgroup analyses were performed according to the type of population (consecutive vs. nonconsecutive) and proportion of mechanically ventilated patients (<50% vs. ≥50%). All meta‐analyses were conducted using the meta package from R 3.6.3 (www.r-project.org). A two‐tailed *p* < .05 was considered statistically significant.

## 3 RESULTS

### 3.1 Study selection

Our electronic search retrieved 807 articles. After the removal of duplicates, 334 articles were screened by title/abstract, and of those, 253 were excluded. After a full‐text assessment of 81 remaining articles, 62 were excluded due to no full‐text (n=2), other population (n=5), conference abstract (n=5), commentary (n=8), other exposure (n=8), and other outcome (n=9). Finally, 19 articles were selected (Supplemental Figure 1).^8‐26^

### 3.2 Study characteristics

The main characteristics of the included studies were summarized in Table 1. A total of 2307 patients were included. The mean age ranged from 59 to 72 years and 65% of patients were male. Most studies were from Italy (*n* = 5) and United Kingdom (*n* = 4). The most common comorbidities were hypertension (51%), diabetes (25%), obesity (22%), and coronary artery disease (14%). The median number of days on which echocardiographic evaluation was performed, ranged from 1 to 8 days after hospital admission. RVFWLS was analyzed using the GE EchoPAC in four studies and the Philips QLAB 13 in two studies. The length of follow‐up varied across studies although was mostly at 30 days and in‐hospital. Three studies^8,12,14^ included only patients hospitalized in the intensive care unit (ICU), while the remaining studies included ICU and non‐ICU patients. The proportion of patients who required mechanical ventilation varied between 4% and 98%. Overall, the mortality ranged from 9% to 44% across studies. Information about the adjusted effect estimates and the included variables in the multivariate analysis are shown in Table S2. The echocardiographic parameters evaluated and the definitions of mortality and RVD can be found in Table S3.

**TABLE 1:** Characteristics of included studies

Study	Country	Sample size	Population	Timing of evaluation^a^	Age^a^	Male	Comorbidities	Mechanical ventilation	Mortality
Bagate, 2021^8^	France	67	Consecutive ICU patients with COVID‐19	1 (1–3) days after ICU admission	61 (50–70)	82%	Hypertension (54%), diabetes (36%), HF (10%), AF (9%)	98%	39%
Bursi, 2020^9^	Italy	49	Hospitalized patients with COVID‐19 who underwent TTE	8 (4–15) days since symptoms onset	65.7 ± 12.6	63%	Hypertension (49%), diabetes (18%), HF (6%), CAD (22%), COPD (12%), AF (8%)	22%	33%
Chen, 2020^10^	USA	143	Consecutive hospitalized patients with COVID‐19	NR	67 ± 16	62%	Hypertension (69%), diabetes (38%), CAD (30%), CKD (19%)	35%	28%
Crook, 2021^11^	UK	27	Hospitalized patients with COVID‐19 who required a TTE	NR	64.5 (37–80)	73%	Hypertension (43%), diabetes (20%), HF (7%), CAD (3%), stroke (7%)	67%	26%
D'Alto, 2020^12^	Italy	94	Hospitalized patients with ARDS and COVID‐19	3 (range 1–7 days) after admission	63.6 ± 12.7	74%	Hypertension (67%), diabetes (17%), CAD (18%), obesity (33%)	39%	26%
D'Andrea, 2020^13^	Italy	89	Consecutive hospitalized patients with COVID‐19	NR	64.6 (range: 20–88)	60%	NR	9%	18%
Gonzalez, 2020^14^	Spain	52	COVID‐19 Patients admitted to the ICU	6 (2–10) days after ICU admission	59.3 ± 13.5	69%	Hypertension (40%), diabetes (29%), CAD (2%), AF (4%), CKD (2%)	88%	15%
Kim, 2020^15^	USA	268	Consecutive hospitalized patients who underwent TTE	6 (1–15) days after admission	65.1 ± 14.1	62%	Hypertension (65%), diabetes (39%), CAD (20%), obesity (32%), COPD (7%)	54%	NR
Lassen, 2020^16^	Denmark	214	Consecutive hospitalized patients with COVID‐19	4 (2–8) days after admission	68.9 ± 13.5	55%	Hypertension (57%), diabetes (25%), hyperlipidemia (40%), HF (10%), CAD (16%), COPD (15%)	NR	12%
Li 1, 2020^17^	China	157	Consecutive hospitalized patients with COVID‐19	NR	62.3 ± 13.1	50%	Hypertension (45%), diabetes (15%), obesity (15%), COPD (6%), CAD (17%), CKD (2%)	24%	15%
Li 2, 2020^18^	China	120	Consecutive hospitalized patients with COVID‐19	7 (3–10) days after admission	61 ± 14	48%	Hypertension (40%), diabetes (12%), obesity (18%), COPD (5%), CAD (9%), CKD (14%)	12%	15%
Mahmoud, 2020^19^	UK	74	Hospitalized patients with COVID‐19 who underwent TTE	5 (3–10) days after admission	59 ± 13	78%	Hypertension (42%), diabetes (36%), CKD (11%), stroke (7%), CAD (9%)	82%	38%
Moody, 2020^20^	UK	164	Hospitalized patients with COVID‐19 who underwent TTE	3 (2–5) days	61 ± 13	78%	Hypertension (41%), diabetes (32%), CKD (12%), stroke (7%), CAD (13%)	73%	40%
Pagnesi, 2020^21^	Italy	200	Consecutive hospitalized non‐ICU patients with COVID‐19	NR	62 (55–74)	65%	Hypertension (42%), diabetes (18%), CAD (7%), HF (3%), dyslipidemia (22%), COPD (5%)	4%	9%
Rath, 2020^22^	Germany	123	Consecutive hospitalized patients with COVID‐19	Within 24 h after admission	68 ± 15	63%	Hypertension (70%), diabetes (24%), dyslipidemia (37%), obesity (19%), CKD (11%), CAD (23%)	40%	13%
Rothschild, 2020^23^	Israel	100	Consecutive hospitalized patients with COVID‐19 who underwent speckle‐tracking TTE	Within 24 h after admission	64.3 ± 20.7	64%	NR	19%	23%
Stockenhuber, 2020^24^	UK	34	Consecutive hospitalized patients with COVID‐19 who underwent TTE	NR	72 ± 2.6	79%	Hypertension (53%), diabetes (35%), CKD (32%), CAD (9%), stroke (9%)	32%	44%
Szekely, 2020^25^	Israel	200	Consecutive hospitalized patients with COVID‐19	Within 24 h after admission	64.2 ± 19.2	60%	Hypertension (54%), diabetes (28%), CAD (15%), HF (11%), stroke (10%), COPD (6%), obesity (24%)	21%	14%
Xie, 2020^26^	China	132	Consecutive hospitalized patients with COVID‐19	7 (3–11) days after admission	61 ± 13	51%	Hypertension (44%), diabetes (11%), obesity (15%), COPD (4%), CAD (14%), CKD (1%)	24%	14%

Abbreviations: CAD, coronary artery disease; COPD, chronic obstructive pulmonary disease; CKD, chronic kidney disease; COVID‐19, coronavirus disease 2019; HF, heart failure; ICU, intensive care unit; NR, not reported; TTE, transthoracic echocardiography; USA, United States of America; UK, United Kingdom.

a Data are mean ± SD or median (interquartile range).

### 3.3 Risk of bias assessment

According to the NOS assessment, 10 studies had a moderate risk of bias and nine studies had a low risk of bias (Table S4). None of the studies was scored as high risk of bias.

### 3.4 Tricuspid annular plane systolic excursion

**FIGURE 1:**
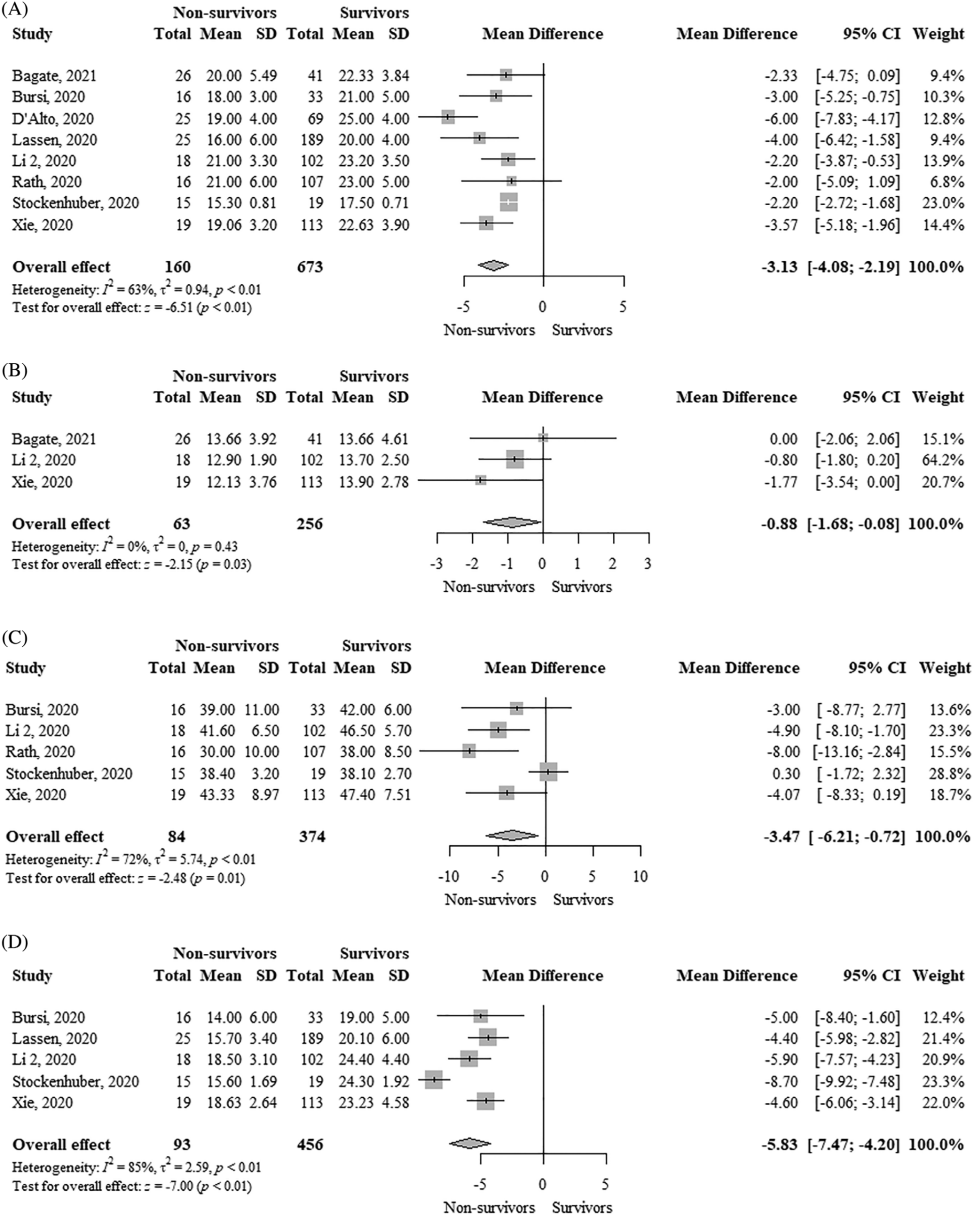
Forest plot showing the comparison of (A) Tricuspid annular plane systolic excursion (mm), (B) Tricuspid S′ peak systolic velocity (cm/s), (C) Fractional area change (%), and (D) Right ventricular free wall longitudinal strain (%) between survivors and nonsurvivors with COVID‐19. COVID‐19, coronavirus disease 2019

**FIGURE 2:**
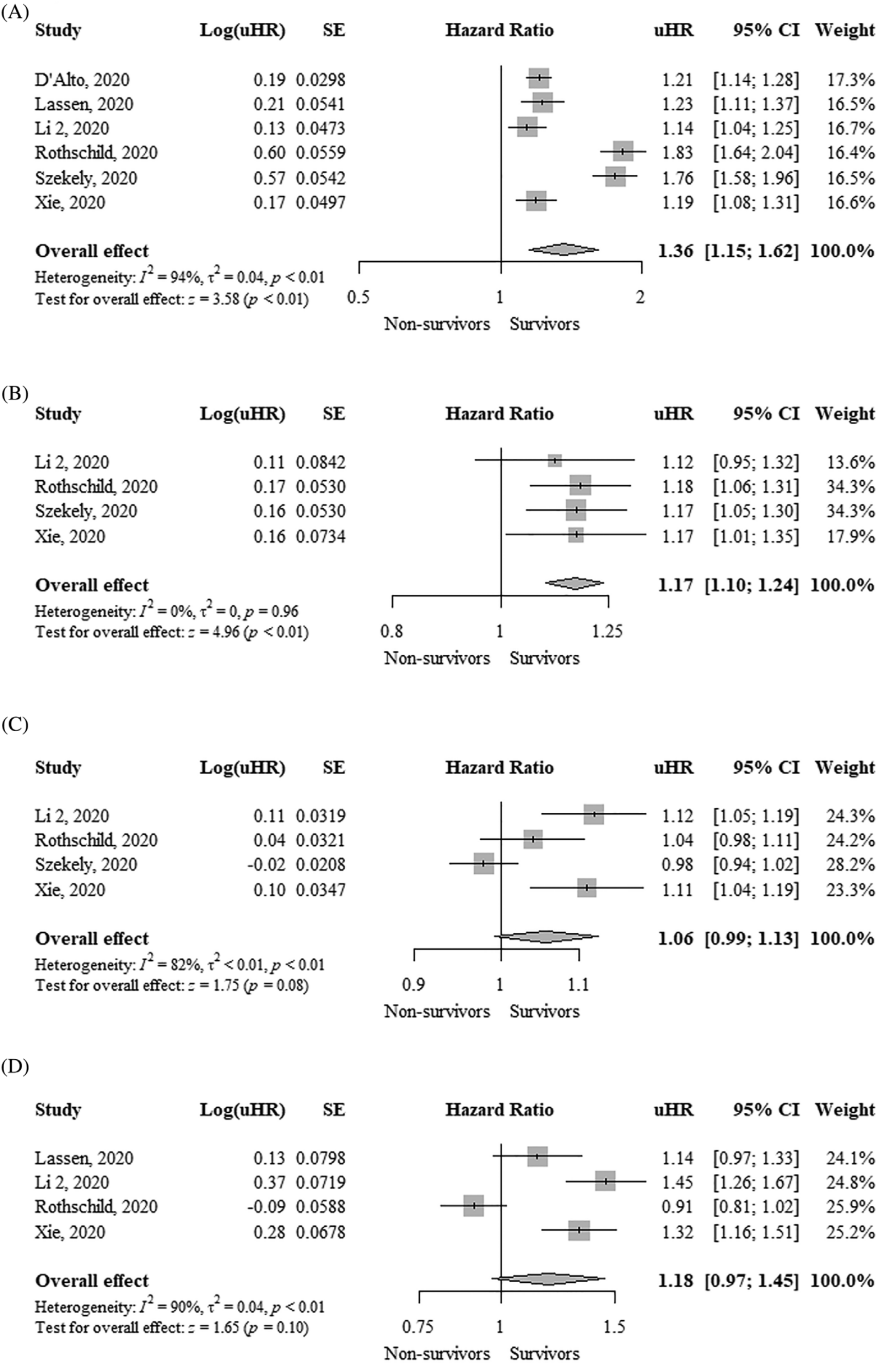
Forest plot showing the association between one unit change in (A) Tricuspid annular plane systolic excursion (mm), (B) Tricuspid S′ peak systolic velocity (cm/s), (C) Fractional area change (%), and (D) Right ventricular free wall longitudinal strain (%) and mortality in COVID‐19 patients (unadjusted estimates). CI, confidence interval; COVID‐19, coronavirus disease 2019; uHR, unadjusted hazard ratio

**FIGURE 3:**
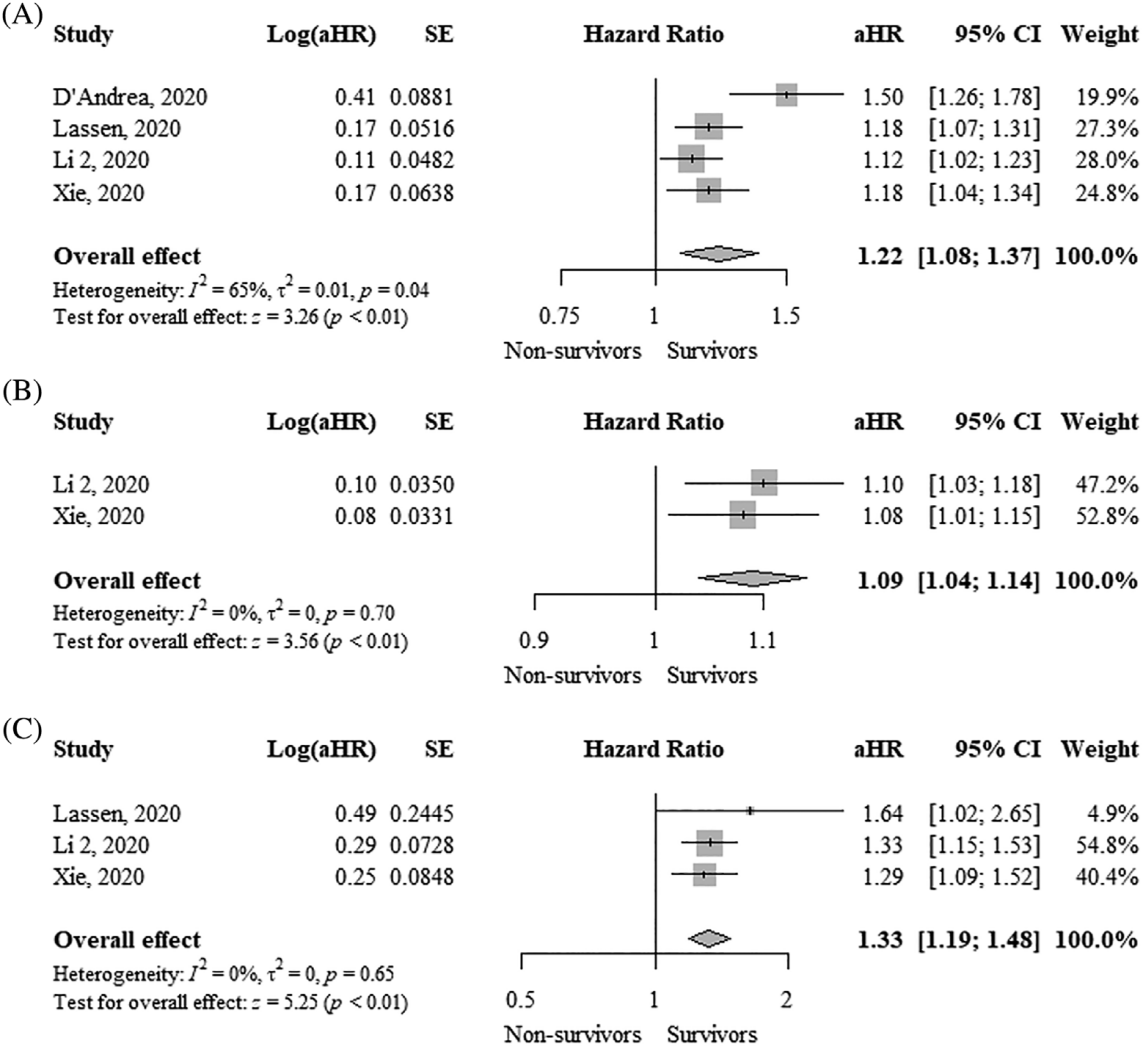
Forest plot showing the association between one unit change in (A) Tricuspid annular plane systolic excursion (mm), (B) Fractional area change (%), and (C) Right ventricular free wall longitudinal strain (%) and mortality in COVID‐19 patients (adjusted estimates). aHR, adjusted hazard ratio; CI, confidence interval; COVID‐19, coronavirus disease 2019

TAPSE was significantly lower (MD, −3.13 mm; 95% CI, −4.08 to −2.19; I^2^ = 63%) in nonsurvivors compared to survivors (Figure 1A). Each 1 mm decrease in TAPSE was significantly associated with a higher mortality using both unadjusted hazard ratio (uHR, 1.36; 95% CI, 1.15–1.62; I^2^ = 94%) and adjusted hazard ratio (aHR, 1.22; 95% CI, 1.08–1.37; I^2^ = 65%) (Figure 2A and Figure 3A). Funnel plots did not show asymmetry (Figure S2).

### 3.5 Tricuspid S′ peak systolic velocity

Tricuspid S′ peak systolic velocity was significantly lower (MD, −0.88 cm/s; 95% CI, −1.68 to −0.08; I^2^ = 0%) in nonsurvivors compared to survivors (Figure 1B). Funnel plot did not show asymmetry (Figure S3). Each 1 cm/s decrease in tricuspid S′ peak systolic velocity was significantly associated with higher mortality (uHR, 1.17; 95% CI, 1.10–1.24; I^2^ = 0%) (Figure 2B). Funnel plot showed asymmetry (Figure S3).

### 3.6 Fractional area change

FAC was significantly lower (MD, −3.47%; 95% CI, −6.21 to −0.72; I^2^ = 72%) in nonsurvivors compared to survivors (Figure 1C). FAC was not significantly associated with higher mortality using an uHR (1.06; 95% CI, 0.99–1.13; I^2^ = 82%) (Figure 2C). In contrast, the adjusted hazard ratio (aHR, 1.09; 95% CI, 1.04–1.14; I^2^ = 0%) showed that each 1% decrease in FAC was significantly associated with higher mortality (Figure 3B). Funnel plots did not show asymmetry (Figure S4).

### 3.7 Right ventricular free wall longitudinal strain

RVFWLS was significantly lower (MD, −5.83%; 95% CI, −7.47 to −4.20; I^2^ = 85%) in survivors compared to nonsurvivors (Figure 1D). RVFWLS was not significantly associated with higher mortality using aHR (1.18; 95% CI, 0.97–1.45; I^2^ = 90%) (Figure 2D). In contrast, the adjusted hazard ratio (aHR, 1.33; 95% CI, 1.19–1.48; I^2^ = 0%) showed that each 1% increase of RVFWLS was significantly associated with higher mortality (Figure 3C). Funnel plots did not show asymmetry (Figure S5).

### 3.8 Right ventricular dysfunction

**FIGURE 4:**
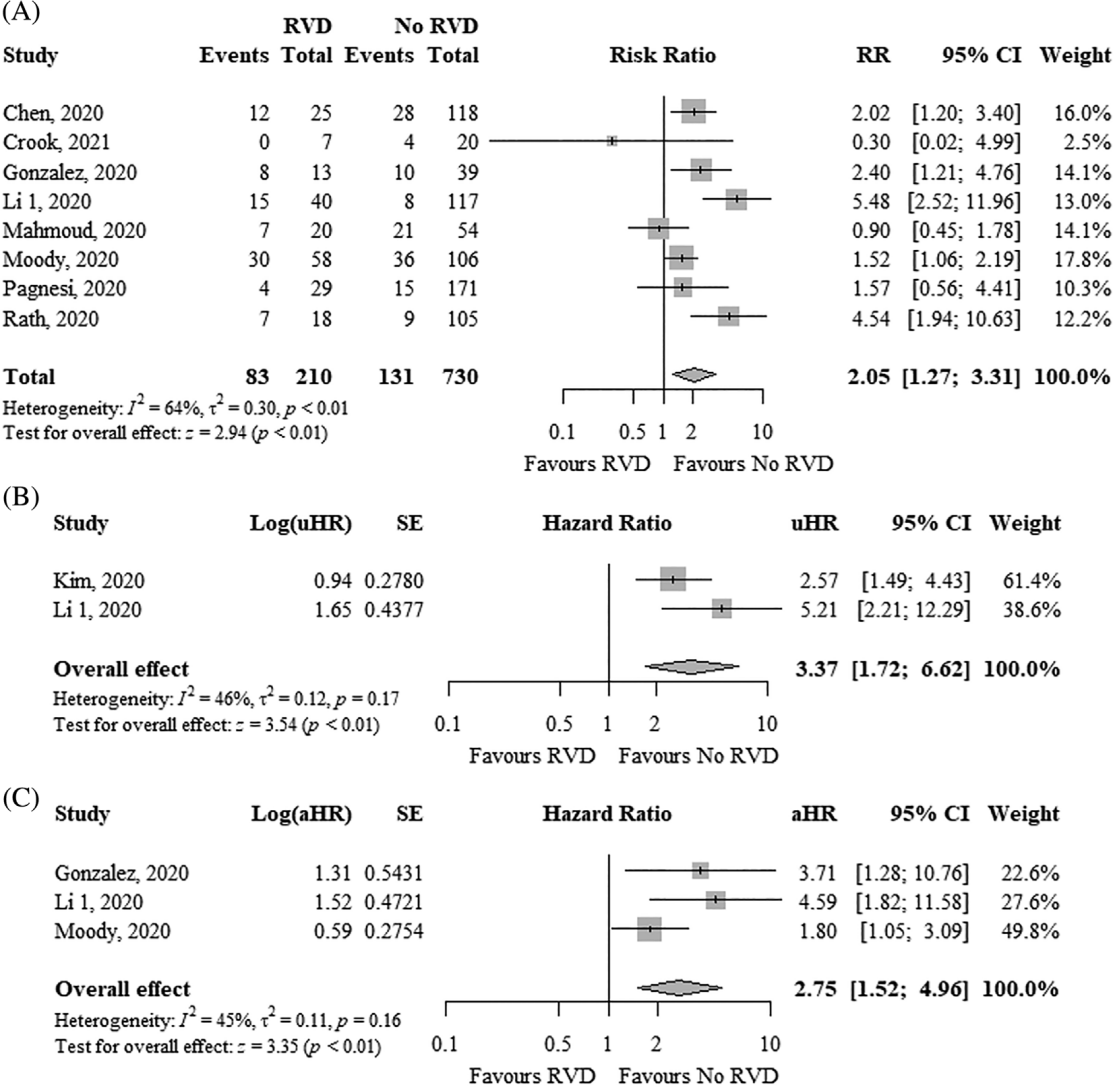
Forest plot showing the (A) RR, (B) uHR, and (C) aHR between right ventricular dysfunction and mortality in COVID‐19 patients. aHR, adjusted hazard ratio; CI, confidence interval; COVID‐19, coronavirus disease 2019; RR, risk ratio; uHR, unadjusted hazard ratio

Overall, 22% of patients had RVD and the absolute risk of mortality in patients with RVD was 40% in eight studies. RVD was significantly associated with higher mortality using unadjusted risk ratio (uRR, 2.05; 95% CI, 1.27–3.31; I^2^ = 64%), uHR (3.37; 95% CI, 1.72–6.62; I^2^ = 46%), and adjusted hazard ratio (aHR, 2.75; 95% CI, 1.52–4.96; I^2^ = 45%) (Figure 4). Funnel plots did not show asymmetry (Figure S6).

### 3.9 Subgroup analyses

Subgroup analyses by type of population (consecutive vs. nonconsecutive) and proportion of mechanical ventilation (<50% vs. ≥50%) showed that RVD was significantly associated with an increased risk of mortality only in studies with consecutive samples (Figure S7) and studies with <50% of mechanically ventilated patients (Figure S8), respectively.

## 4 DISCUSSION

The current study demonstrates that abnormalities in the echocardiographic markers of RVD, namely, TAPSE, tricuspid S′ peak systolic velocity, FAC, and RVFWLS, were significantly associated with poor survival in patients with COVID‐19. Specifically, each 1 mm decrease in TAPSE, each 1% decrease of FAC, and each 1% increase of RVFWLS were independently associated with higher mortality. Likewise, RVD was significantly associated with higher mortality using unadjusted and adjusted outcome measures. The risk of bias was low or moderate across the studies.

Although it is well known that COVID‐19 patients can develop left and right ventricular abnormalities,^27,28^ several reports indicate that RVD occurs more frequently than left ventricular dysfunction in these patients.^19,29,30^ Furthermore, SARS‐CoV‐2 can cause pulmonary damage at different levels (vasculature, parenchyma, and interstitium) leading to patients requiring mechanical ventilation. These unique pathophysiological alterations place the right ventricle at high risk for failure in COVID‐19 patients.

RVD is a known prognostic factor in several cardiovascular disorders such as heart failure, pulmonary hypertension, and valvular heart disease.^31^ However, its role in systemic viral infections such as SARS‐CoV‐2 is less known.^3^ Recently, there are several single‐center studies with small sample sizes that have evaluated the impact of RVD on the prognosis of patients with COVID‐19. The largest published study was a multicenter registry that included 268 hospitalized patients with COVID‐19 who underwent transthoracic echocardiography.^15^ This study found that right ventricular dilatation and dysfunction independently increased the risk of mortality. In addition, the adverse right ventricular remodeling (dilatation or dysfunction) provided incremental risk stratification beyond clinical‐ and biomarker‐based assessments. Interestingly, RVD occurred less frequently than dilatation and was present mainly in cases with greater dilatation, suggesting that probably dilatation is the initial response to the myocardial insult that ultimately results in RVD. We found that RVD was independently associated with an almost threefold increase in mortality in COVID‐19 patients. Overall, our finding highlights the possible prognostic value of the right ventricular function assessment that can potentially improve the risk stratification of these patients.

How RVD impacts clinical outcomes in COVID‐19 patients remains incompletely understood. RVD may contribute to the rapid hemodynamic deterioration of these patients by affecting the left ventricular forward stroke volume.^31^ Another possible explanation is that the presence of myocardial damage will provide the pathological substrate for malignant ventricular arrhythmias, predisposing to sudden cardiac death. However, although we found that RVD increases the risk of death in COVID‐19 patients, it is also possible the option of a common cause for both problems.

In the context of COVID‐19, the two main pathophysiological mechanisms that may explain RVD include increased afterload and impaired contractility.^32^ Although both mechanisms can coexist, the first mechanism seems to predominate because the right ventricle is highly sensitive to changes in afterload.

There are some clinical conditions associated with SARS‐CoV‐2 infection that can lead to increased afterload such as pulmonary thromboembolism, acute respiratory distress syndrome, and mechanical ventilation in intubated patients.^32^ Many studies have shown an elevated incidence of pulmonary thromboembolism in COVID‐19 patients.^33^ Likewise, several reports are showing that these patients exhibit marked alterations of the coagulation pathways as evidence by increased levels of D‐dimer, fibrinogen, prothrombin time, and activated partial thromboplastin time.^34^ A recent autopsy study of patients who died of COVID‐19 showed a higher incidence of deep vein thrombosis, pulmonary embolism, and microthrombi formation within the small to the mid‐sized pulmonary vasculature.^35^ This prothrombotic state can be explained by the interaction of several pathophysiological mechanisms. The initial response provoked by the SARS‐CoV‐2 infection is characterized by the release of pro‐inflammatory cytokines such as IL‐1β, IL‐6, and TNF‐α which, in turn, induces the activation of pro‐coagulant factors and platelets, impairs fibrinolysis, and promotes the endothelial damage.^36^ In addition, endothelial cells are an important viral target by expressing the ACE2 receptor. Reduced ACE2 expression may lead to an imbalance of the renin‐angiotensin‐aldosterone system favoring the pro‐coagulant and vasoconstrictor effects mediated by angiotensin II.^36^

Acute respiratory distress syndrome is a major cause of death in COVID‐19 patients with an incidence of up to 68% in hospitalized patients.^37^ RVD is an independent predictor of mortality in ARDS patients and its development is attributed to the increased pulmonary vascular resistance, which is mediated by vascular remodeling, right ventricular–pulmonary arterial uncoupling, and thrombosis/vasoconstriction of the pulmonary vasculature.^38^

Mechanical ventilation, especially when high levels of positive end‐expiratory pressure are required, reduces venous return and increases pulmonary vascular resistance.^39^ Considering that up to 33% of hospitalized patients with COVID‐19 are mechanically ventilated,^40^ the potential damage that this invasive therapy may cause to the right ventricle should be considered in all cases. Thus, the management of this equipment should be adequately optimized in order to reduce the risk of RVD.

Currently, it is recognized that several cardiac cells such as cardiomyocytes, pericytes, and fibroblasts can also express the ACE2 receptor and are therefore susceptible to infection by SARS‐CoV‐2.^36^ Many cases of acute myocarditis in patients with COVID‐19 have been described in the literature and it is considered a cause of right and/or left heart failure.^41^ Interestingly, there are published autopsy reports that have shown lymphocytic infiltrates in the right ventricular myocardium of these patients.^35^ Overall, it appears that direct viral damage, exacerbated systemic inflammatory response, and other pathogenic mechanisms such as epicardial and microvascular coronary artery disease secondary to thrombosis also contribute to the development of ventricular dysfunction in COVID‐19 patients.^27^

The assessment of right ventricular systolic function has historically been a difficult task given its complex geometric shape. Although echocardiography has some limitations, it remains the most commonly used imaging tool worldwide.^42^ TAPSE, tricuspid S′ peak systolic velocity, and FAC are the most frequently used echocardiographic parameters to evaluate right ventricular systolic function in daily clinical practice.^42,43^ Of note, these parameters can assess both longitudinal (e.g., TAPSE and tricuspid S′ peak systolic velocity) and radial function (e.g., FAC) of the right ventricle.^44^ More recent techniques such as speckle‐tracking echocardiography are increasingly used to estimate the global and regional systolic function.^43^ This technique allows evaluating the longitudinal myocardial deformation of the right ventricle, particularly of the free wall (e.g., RVFWLS). Among these four echocardiographic parameters, our results indicate that TAPSE, FAC, and RVFWLS were significantly associated with mortality in COVID‐19 patients in adjusted models. Overall, these findings suggest that both one‐ and two‐dimensional parameters have a prognostic value. Right ventricular ejection fraction by three‐dimensional echocardiography is another parameter that evaluates the global right ventricular systolic function. Unfortunately, none of the included studies in our study used this measure, although it seems to be more accurate and with better reproducibility compared to other parameters. Nevertheless, the assessment of right ventricular function by three‐dimensional echocardiography needs a very good acoustic. This parameter is quite difficult to obtain in ICU patients, particularly when ventilated and with often a poor acoustic window.

Our study has some limitations. First, heterogeneity was high in several pooled estimates. Possible reasons for heterogeneity include sample size, the definition of RVD, type of population, time of follow‐up, and type of echocardiogram analysts. Second, data on the prevalence of myocarditis and acute pulmonary thromboembolism was not reported in included studies, limiting the applicability of the proposed pathophysiological hypothesis. Third, since almost half of the included studies were conducted in only two countries (Italy and the United Kingdom), our results are not necessarily generalizable to other regions with different mortality rates. Fourth, the time from hospital admission to echocardiogram performance varied among studies with a median ranging from 1 day to 8 days and being performed in a quarter of the studies after approximately 1 week. This variation in echocardiogram time could influence the likelihood of developing RVD as patients could worsen during hospitalization, potentially introducing selection bias. Fifth, TAPSE and tricuspid S′ peak systolic velocity only reflects basal right ventricular function. In clinical conditions with regional dysfunction such as pulmonary thromboembolism, which has been reported in many COVID‐19 patients, both measures may underestimate global right ventricular function. Therefore, the prevalence of RVD may be even higher in the COVID‐19 setting. Finally, since most of the included studies did not report adjusted effects, there is an increased risk of bias in their pooled estimates. Although we also reported meta‐analyses of adjusted estimates of available studies, there is still a possibility of residual confounding. Thus, our results should be interpreted with caution.

## 5 CONCLUSION

Our review shows that RVD was associated with an increased risk of mortality in COVID‐19 patients. Among echocardiographic parameters, TAPSE, FAC, and RVFWLS were independently associated with higher mortality. Although our results need to be confirmed by prospective studies with larger sample sizes, they suggest that echocardiographic assessment of right ventricular systolic function may add prognostic information during the risk stratification of COVID‐19 patients.

## CONFLICT OF INTEREST

None of the authors reported any conflicts of interest.

## AUTHOR CONTRIBUTIONS

Carlos Diaz‐Arocutipa and Jose Saucedo‐Chinchay involved in concept/design. Carlos Diaz‐Arocutipa and Jose Saucedo‐Chinchay involved in data acquisition. Carlos Diaz‐Arocutipa, Jose Saucedo‐Chinchay, and Edgar Argulian involved in data analysis/interpretation. Carlos Diaz‐Arocutipa drafted the article. Jose Saucedo‐Chinchay and Edgar Argulian critically revised the article. Carlos Diaz‐Arocutipa, Jose Saucedo‐Chinchay, and Edgar Argulian approved the article.

## Supporting information


**Appendix S1:** Supporting Information

## Data Availability

The data that support the findings of this study are available from the corresponding author upon reasonable request.

## References

[1] WHO Coronavirus Disease (COVID‐19) Dashboard. https://covid19.who.int/ 2021.

[2] Nishiga M , Wang DW , Han Y , Lewis DB , Wu JC . COVID‐19 and cardiovascular disease: from basic mechanisms to clinical perspectives. Nat Rev Cardiol. 2020;17(9):543‐558. 32690910 10.1038/s41569-020-0413-9 PMC7370876

[3] Lan Y , Liu W , Zhou Y . Right ventricular damage in COVID‐19: association between myocardial injury and COVID‐19. Front Cardiovasc Med. 2021;8:606318. 33665210 10.3389/fcvm.2021.606318 PMC7920943

[4] Moher D , Liberati A , Tetzlaff J , Altman DG . Preferred reporting items for systematic reviews and meta‐analyses: the PRISMA statement. BMJ. 2009;339:b2535. 19622551 10.1136/bmj.b2535 PMC2714657

[5] G. Wells , B. Shea , D. O'Connell , J. Peterson , V. Welch , M. Losos and P. Tugwell . The Newcastle‐Ottawa scale (NOS) for assessing the quality of nonrandomised studies in meta‐analysis, 2021, http://www.ohri.ca/programs/clinical_epidemiology/oxfordasp.

[6] Langan D , Higgins JPT , Simmonds M . Comparative performance of heterogeneity variance estimators in meta‐analysis: a review of simulation studies. Res Synth Methods. 2017;8(2):181‐198. 27060925 10.1002/jrsm.1198

[7] Wan X , Wang W , Liu J , Tong T . Estimating the sample mean and standard deviation from the sample size, median, range and/or interquartile range. BMC Med Res Methodol. 2014;14:135. 25524443 10.1186/1471-2288-14-135 PMC4383202

[8] Bagate F , Masi P , d'Humières T , et al. Advanced echocardiographic phenotyping of critically ill patients with coronavirus‐19 sepsis: a prospective cohort study. J Intensive Care Med. 2021;9(1). 10.1186/s40560-020-00516-6 PMC7816136 33472693

[9] Bursi F , Santangelo G , Sansalone D , et al. Prognostic utility of quantitative offline 2D‐echocardiography in hospitalized patients with COVID‐19 disease. Echocardiography. 2020;37(12):2029‐2039. 32964483 10.1111/echo.14869 PMC7646664

[10] Chen LQ , Burdowski J , Marfatia R , et al. Reduced cardiac function is associated with cardiac injury and mortality risk in hospitalized COVID‐19 patients. Clin Cardiol. 2020;43(12):1547‐1554. 33280140 10.1002/clc.23479 PMC7675371

[11] Crook RL , Williams H , Green M , et al. Prospective multicentre cohort study of transthoracic echocardiography provision in the south west of the UKduring the first wave of SARS‐CoV‐2 pandemic. Open Heart. 2021;8(1):e001409. 33504630 10.1136/openhrt-2020-001409 PMC7843208

[12] D'Alto M , Marra AM , Severino S , et al. Right ventricular‐arterial uncoupling independently predicts survival in COVID‐19 ARDS. Crit Care. 2020;24(1):670. 33256813 10.1186/s13054-020-03385-5 PMC7703719

[13] D'Andrea A , Scarafile R , Riegler L , et al. Right ventricular function and pulmonary pressures as independent predictors of survival in patients with COVID‐19 pneumonia. JACC Cardiovasc Imaging. 2020;13(11):2467‐2468. 32654965 10.1016/j.jcmg.2020.06.004 PMC7314435

[14] Gonzalez‐Fernandez O , Ponz de Antonio I , Rosillo Rodriguez SO , Ruiz Cantador J , Figueira Iglesias JC , Lopez‐Sendon Hentschel JL . D‐dimer and right ventricular abnormalities as prognostic factors in critically ill COVID‐19 patients. Rev Esp Cardiol. 2020;73(11):966‐968. 32798152 10.1016/j.rec.2020.07.004 PMC7365129

[15] Kim J , Volodarskiy A , Sultana R , et al. Prognostic utility of right ventricular remodeling over conventional risk stratification in patients with COVID‐19. J Am Coll Cardiol. 2020;76(17):1965‐1977. 33092732 10.1016/j.jacc.2020.08.066 PMC7572068

[16] Lassen MCH , Skaarup KG , Lind JN , et al. Echocardiographic abnormalities and predictors of mortality in hospitalized COVID‐19 patients: the ECHOVID‐19 study. ESC Heart Fail. 2020;7(6):4189‐4197. 10.1002/ehf2.13044 PMC7755011 33089972

[17] Li Y , Li H , Li M , Zhang L , Xie M . The prevalence, risk factors and outcome of cardiac dysfunction in hospitalized patients with COVID‐19. Intensive Care Med. 2020;46(11):2096‐2098. 32767075 10.1007/s00134-020-06205-0 PMC7411264

[18] Li Y , Li H , Zhu S , et al. Prognostic value of right ventricular longitudinal strain in patients with COVID‐19. JACC Cardiovasc Imaging. 2020;13(11):2287‐2299. 32654963 10.1016/j.jcmg.2020.04.014 PMC7195441

[19] Mahmoud‐Elsayed HM , Moody WE , Bradlow WM , et al. Echocardiographic findings in patients with COVID‐19 pneumonia. Can J Cardiol. 2020;36(8):1203‐1207. 32474111 10.1016/j.cjca.2020.05.030 PMC7255734

[20] Moody WE , Mahmoud‐Elsayed HM , Senior J , et al. Impact of right ventricular dysfunction on mortality in patients hospitalized with COVID‐19. CJC Open. 2021;3(1):91‐100. 32984798 10.1016/j.cjco.2020.09.016 PMC7502228

[21] Pagnesi M , Baldetti L , Beneduce A , et al. Pulmonary hypertension and right ventricular involvement in hospitalised patients with COVID‐19. Heart. 2020;106(17):1324. 32675217 10.1136/heartjnl-2020-317355 PMC7476272

[22] Rath D , Petersen‐Uribe Á , Avdiu A , et al. Impaired cardiac function is associated with mortality in patients with acute COVID‐19 infection. Clin Res Cardiol. 2020;109(12):1491‐1499. 32537662 10.1007/s00392-020-01683-0 PMC7293880

[23] Rothschild E , Baruch G , Szekely Y , et al. The predictive role of left and right ventricular speckle‐tracking echocardiography in COVID‐19. JACC Cardiovasc Imaging. 2020;13(11):2471‐2474. 33011117 10.1016/j.jcmg.2020.07.026 PMC7434478

[24] Stockenhuber A , Vrettos A , Androschuck V , et al. A pilot study on right ventricular longitudinal strain as a predictor of outcome in COVID‐19 patients with evidence of cardiac involvement. Echocardiography. 2021;38:222–229. 33368601 10.1111/echo.14966

[25] Szekely Y , Lichter Y , Hochstadt A , et al. The predictive role of combined cardiac and lung ultrasound in Coronavirus Disease. J Am Soc Echocardiogr. 2021;34:642–652. 33571647 10.1016/j.echo.2021.02.003 PMC7870445

[26] Xie Y , Wang L , Li M , et al. Biventricular longitudinal strain predict mortality in COVID‐19 patients. Front Cardiovasc Med. 2020;7:632434. 33537350 10.3389/fcvm.2020.632434 PMC7848071

[27] Bieber S , Kraechan A , Hellmuth JC , et al. Left and right ventricular dysfunction in patients with COVID‐19‐associated myocardial injury. Infection. 2021;49(3):491‐500. 33515390 10.1007/s15010-020-01572-8 PMC7846912

[28] Soulat‐Dufour L , Fauvel C , Weizman O , et al. Prognostic value of right ventricular dilatation in patients with COVID‐19: a multicentre study. Eur Heart J Cardiovasc Imaging. 2021. 10.1093/ehjci/jeab067 PMC8600376 34008835

[29] Silverio A , Di Maio M , Scudiero F , et al. Clinical conditions and echocardiographic parameters associated with mortality in COVID‐19. Eur J Clin Invest. 2021;e13638. 34287861 10.1111/eci.13638 PMC8420215

[30] Szekely Y , Lichter Y , Taieb P , et al. Spectrum of cardiac manifestations in COVID‐19: a systematic echocardiographic study. Circulation. 2020;142(4):342‐353. 32469253 10.1161/CIRCULATIONAHA.120.047971 PMC7382541

[31] Sanz J , Sánchez‐Quintana D , Bossone E , Bogaard HJ , Naeije R . Anatomy, function, and dysfunction of the right ventricle: JACC state‐of‐the‐art review. J Am Coll Cardiol. 2019;73(12):1463‐1482. 30922478 10.1016/j.jacc.2018.12.076

[32] Park JF , Banerjee S , Umar S . In the eye of the storm: the right ventricle in COVID‐19. Pulm Circ. 2020;10(3):2045894020936660. 32655856 10.1177/2045894020936660 PMC7333504

[33] Malas MB , Naazie IN , Elsayed N Mathlouthi A Marmor R Clary B . Thromboembolism risk of COVID‐19 is high and associated with a higher risk of mortality: a systematic review and meta‐analysis. EClinicalMedicine. 2020;29:100639. 33251499 10.1016/j.eclinm.2020.100639 PMC7679115

[34] Luo HC , You CY , Lu SW , Fu YQ . Characteristics of coagulation alteration in patients with COVID‐19. Ann Hematol. 2021;100(1):45‐52. 33079220 10.1007/s00277-020-04305-x PMC7572245

[35] Wichmann D , Sperhake JP , Lütgehetmann M , et al. Autopsy findings and venous thromboembolism in patients with COVID‐19: a prospective cohort study. Ann Intern Med. 2020;173(4):268‐277. 32374815 10.7326/M20-2003 PMC7240772

[36] Bonnemain J , Ltaief Z , Liaudet L . The right ventricle in COVID‐19. J Clin Med. 2021;10(12):2035. 34200990 10.3390/jcm10122535 PMC8230058

[37] Tzotzos SJ , Fischer B , Fischer H Zeitlinger M . Incidence of ARDS and outcomes in hospitalized patients with COVID‐19: a global literature survey. Crit Care. 2020;24(1):516. 32825837 10.1186/s13054-020-03240-7 PMC7441837

[38] Zochios V , Parhar K , Tunnicliffe W , Roscoe A Gao F . The right ventricle in ARDS. Chest. 2017;152(1):181‐193. 28267435 10.1016/j.chest.2017.02.019

[39] Disselkamp M , Adkins D , Pandey S , Coz Yataco AO . Physiologic approach to mechanical ventilation in right ventricular failure. Ann Am Thorac Soc. 2018;15(3):383‐389. 29493338 10.1513/AnnalsATS.201707-533CC

[40] Wunsch H . Mechanical ventilation in COVID‐19: interpreting the current epidemiology. Am J Respir Crit Care Med. 2020;202(1):1‐4. 32402207 10.1164/rccm.202004-1385ED PMC7328308

[41] Castiello T Georgiopoulos G Finocchiaro G , et al. COVID‐19 and myocarditis: a systematic review and overview of current challenges. Heart Fail Rev. 2021;1‐11. 33761041 10.1007/s10741-021-10087-9 PMC7988375

[42] Schneider M , Aschauer S , Mascherbauer J , et al. Echocardiographic assessment of right ventricular function: current clinical practice. Int J Cardiovasc Imaging. 2019;35(1):49‐56. 30191507 10.1007/s10554-018-1428-8 PMC6373282

[43] Lang RM , Badano LP , Mor‐Avi V , et al. Recommendations for cardiac chamber quantification by echocardiography in adults: an update from the American Society of Echocardiography and the European Association of Cardiovascular Imaging. J Am Soc Echocardiogr. 2015;28(1):1‐39.e14. 25559473 10.1016/j.echo.2014.10.003

[44] Zaidi A , Knight DS , Augustine DX , et al. Echocardiographic assessment of the right heart in adults: a practical guideline from the British Society of Echocardiography. Echo Res Pract. 2020;7(1):G19‐g41. 32105053 10.1530/ERP-19-0051 PMC7077526

