# Supplementary material for: Association between right ventricular dysfunction and mortality in COVID‐19 patients: A systematic review and meta‐analysis
Source: Clin Cardiol. 2021 Sep 16;44(10):1360–70. doi: 10.1002/clc.23719 (PMC8495092; doi:10.1002/clc.23719)
Supplement: Supplementary file 1 — Appendix S1: Supporting Information [file CLC-44--s001.doc]

**SUPPLEMENTARY DATA**

**Supplemental Table 1. Search strategy**

| **PubMed**  ("right ventricle"[tiab] OR "right ventricular"[tiab] OR "right-sided failure"[tiab] OR "right-sided heart failure"[tiab] OR "right heart"[tiab] OR "right systolic"[tiab]) AND ("Severe Acute Respiratory Syndrome Coronavirus 2"[tiab] OR (("coronavirus"[tiab] OR "corona virus"[tiab] OR "coronaviruses"[tiab]) AND ("wuhan"[tiab] OR "outbreak"[tiab] OR "pandemic"[tiab] OR "2019"[tiab] OR "19"[tiab] OR "novel"[tiab] OR "new"[tiab]) AND "2020"[all]) OR "2019-nCoV"[tiab] OR "nCoV-2019"[tiab] OR "COVID 2019"[tiab] OR "COVID19"[tiab] OR "COVID-19"[tiab] OR "SARS-CoV2"[tiab] OR "SARS-CoV-2"[tiab] OR "SARSCoV-2"[tiab]) |
| --- |
| **Embase**  ('right ventricle'/exp OR 'right ventricle' OR 'right ventricular' OR 'right-sided failure' OR 'right-sided heart failure' OR 'right heart'/exp OR 'right heart' OR 'right systolic') AND ('severe acute respiratory syndrome coronavirus 2'/exp OR 'severe acute respiratory syndrome coronavirus 2' OR (('coronavirus'/exp OR 'coronavirus' OR 'corona virus'/exp OR 'corona virus' OR 'coronaviruses') AND ('wuhan'/exp OR 'wuhan' OR 'outbreak'/exp OR 'outbreak' OR 'pandemic'/exp OR 'pandemic' OR '2019' OR '19' OR 'novel' OR 'new') AND '2020') OR '2019-ncov'/exp OR '2019-ncov' OR 'ncov-2019'/exp OR 'ncov-2019' OR 'covid 2019'/exp OR 'covid 2019' OR 'covid19'/exp OR 'covid19' OR 'covid-19'/exp OR 'covid-19' OR 'sars-cov2' OR 'sars-cov-2'/exp OR 'sars-cov-2' OR 'sarscov-2') |
| **Scopus**  TITLE-ABS-KEY (("right ventricle" OR "right ventricular" OR "right-sided failure" OR "right-sided heart failure" OR "right heart" OR "right systolic") AND (“severe acute respiratory syndrome coronavirus 2” OR ((“coronavirus” OR “corona virus” OR “coronaviruses”) AND (“wuhan” OR “outbreak” OR “pandemic” OR “2019” OR “19” OR “novel” OR “new”) AND “2020”) OR “2019-nCoV” OR “nCoV-2019” OR “COVID 2019” OR “COVID19” OR “COVID-19” OR “SARS-CoV2” OR “SARS-CoV-2” OR “SARSCoV-2”)) |
| **Web of Science**  TS=(("right ventricle" OR "right ventricular" OR "right-sided failure" OR "right-sided heart failure" OR "right heart" OR "right systolic") AND (“severe acute respiratory syndrome coronavirus 2” OR ((“coronavirus” OR “corona virus” OR “coronaviruses”) AND (“wuhan” OR “outbreak” OR “pandemic” OR “2019” OR “19” OR “novel” OR “new”) AND “2020”) OR “2019-nCoV” OR “nCoV-2019” OR “COVID 2019” OR “COVID19” OR “COVID-19” OR “SARS-CoV2” OR “SARS-CoV-2” OR “SARSCoV-2”)) |

**Supplemental Table 2. Adjusted effect estimates of the effect of right ventricular dysfunction on mortality in COVID-19 patients**

| Study | Exposure | Effect estimate | 95% CI | Adjusted variables |
| --- | --- | --- | --- | --- |
| Lassen, 2020 | TAPSE | HR: 0.82 | 0.69-0.93 | Age, sex, hypertension, diabetes, BMI, smoking |
|  | RVFWLS | HR: 1.64 | 1.02-2.66 | Age, sex, hypertension, diabetes, BMI, smoking |
| Li 1, 2020 | Right ventricular dysfunction | HR: 4.59 | 1.82-11.58 | ARDS, troponin, mechanical ventilation |
| Li 2, 2020 | TAPSE | HR: 0.88 | 0.78-0.99 | Sex, ARDS |
|  | FAC | HR: 0.90 | 0.83-0.98 | Sex, ARDS |
|  | RVFWLS | HR: 1.33 | 1.15-1.53 | Sex, ARDS |
| Xie, 2020 | TAPSE | HR: 0.82 | 0.69-0.98 | ARDS, troponin, LV GLS-4ch |
|  | FAC | HR: 0.92 | 0.85-0.99 | ARDS, troponin |
|  | RVFWLS | HR: 1.29 | 1.09-1.52 | ARDS, troponin |
| D'Andrea, 2020 | TAPSE | HR: 0.50 | 0.22-0.74 | Troponin, PaO_2_ at admission, mPAP |
| Gonzalez, 2020 | Right ventricular dysfunction | HR: 3.71 | 1.28-10.76 | Age, D-dimer |
| Moody, 2020 | Right ventricular dysfunction | HR: 1.80 | 1.05-3.09 | Age, sex, diabetes, hypertension, chronic lung disease, malignancy, troponin |

CI, confidence interval; HR, hazard ratio; TAPSE, tricuspid annular plane systolic excursion; FAC, fractional area change; RVFWLS, right ventricular free wall longitudinal strain; BMI, body mass index; ARDS, acute respiratory distress syndrome; LV GLS-4ch, left ventricular global longitudinal strain from the apical four-chamber view; mPAP, mean pulmonary artery pressure; PaO_2_, partial pressure of oxygen.

**Supplemental** Table 3. Definitions of right ventricular dysfunction and mortality

| **Study** | **Echocardiographic parameters** | **Definition of mortality** | **Definition of right ventricular dysfunction** |
| --- | --- | --- | --- |
| Bagate, 2021 | TAPSE, tricuspid S’ peak systolic velocity | 28-day death | NR |
| Bursi, 2020 | TAPSE, FAC, RVFWLS | In-hospital death | NR |
| Chen, 2020 | Right ventricular dysfunction | in-hospital mortality | Visual estimation of the right ventricular ejection fraction <50% |
| Crook, 2021 | Right ventricular dysfunction | In-hospital death | Dilated and impaired right ventricle |
| D'Alto, 2020 | TAPSE | In-hospital death | NR |
| Gonzalez, 2020 | Right ventricular dysfunction | 30-day death | NR |
| Kim, 2020 | Right ventricular dysfunction | In-hospital death | TAPSE <16 mm and tricuspid S’ peak systolic velocity <10 cm/sec |
| Lassen, 2020 | TAPSE, RVFWLS | Death related to COVID-19 | NR |
| Li 1, 2020 | Right ventricular dysfunction | All-cause mortality | Abnormal right ventricular function |
| Li 2, 2020 | TAPSE, tricuspid S’ peak systolic velocity, FAC, RVFWLS | In-hospital death | NR |
| Mahmoud, 2020 | Right ventricular dysfunction | In-hospital death | FAC <35% or TAPSE <17 mm |
| Moody, 2020 | Right ventricular dysfunction | In-hospital death | FAC <35% or TAPSE <17 mm |
| Pagnesi, 2020 | Right ventricular dysfunction | All-cause mortality | TAPSE <17 mm or tricuspid S’ peak systolic velocity <9.5 cm/sec |
| Rath, 2020 | TAPSE, FAC, right ventricular dysfunction | 30-day all-cause mortality | FAC <35% |
| Rothschild, 2020 | TAPSE, tricuspid S’ peak systolic velocity, FAC, RVFWLS | All-cause mortality | NR |
| Stockenhuber, 2020 | TAPSE, FAC, RVFWLS | 30-day death | NR |
| Szekely, 2020 | TAPSE, tricuspid S’ peak systolic velocity, FAC | All-cause mortality | NR |
| Xie, 2020 | TAPSE, tricuspid S’ peak systolic velocity, FAC, RVFWLS | In-hospital death | NR |
| D'Andrea, 2020 | TAPSE | In-hospital death | NR |

TAPSE, tricuspid annular plane systolic excursion; FAC, fractional area change; RVFWLS, right ventricular free wall longitudinal strain; NR, not reported; COVID-19, coronavirus disease 2019.

**Supplemental Table 4. Newcastle-Ottawa scale for risk of bias assessment of cohort studies**

| Study | SELECTION | | | | COMPARABILITY | OUTCOME | | | Total (maximum = 9) |
| --- | --- | --- | --- | --- | --- | --- | --- | --- | --- |
|  | Representativeness of the exposed cohort | Selection of the non-exposed cohort | Ascertainment of the exposure | Outcome status at start of study |  | Assessment of the outcome | Length of follow-up | Adequacy of follow-up |  |
| Bagate, 2021 | * | * | * | * |  | * | * | * | 7 |
| Bursi, 2020 |  | * | * | * |  | * | * | * | 6 |
| Crook, 2021 |  | * | * | * |  | * | * | * | 6 |
| Chen, 2020 |  | * | * | * | ** | * | * | * | 8 |
| D'Alto, 2020 | * | * | * | * |  | * | * | * | 7 |
| Gonzalez, 2020 | * | * | * | * | * | * | * | * | 8 |
| Kim, 2020 |  | * | * | * |  | * | * | * | 6 |
| Lassen, 2020 | * | * | * | * | ** | * | * | * | 9 |
| Li 1, 2020 | * | * | * | * | ** | * | * | * | 9 |
| Li 2, 2020 | * | * | * | * | ** | * | * | * | 9 |
| Mahmoud, 2020 |  | * | * | * |  | * | * | * | 6 |
| Moody, 2020 |  | * | * | * | ** | * | * | * | 8 |
| Pagnesi, 2020 | * | * | * | * |  | * | * | * | 7 |
| Rath, 2020 | * | * | * | * |  | * | * | * | 7 |
| Rothschild, 2020 | * | * | * | * |  | * | * | * | 7 |
| Stockenhuber, 2020 |  | * | * | * |  | * | * | * | 6 |
| Szekely, 2020 | * | * | * | * | * | * | * | * | 8 |
| Xie, 2020 | * | * | * | * | * | * | * | * | 8 |
| D'Andrea, 2020 | * | * | * | * | ** | * | * | * | 9 |


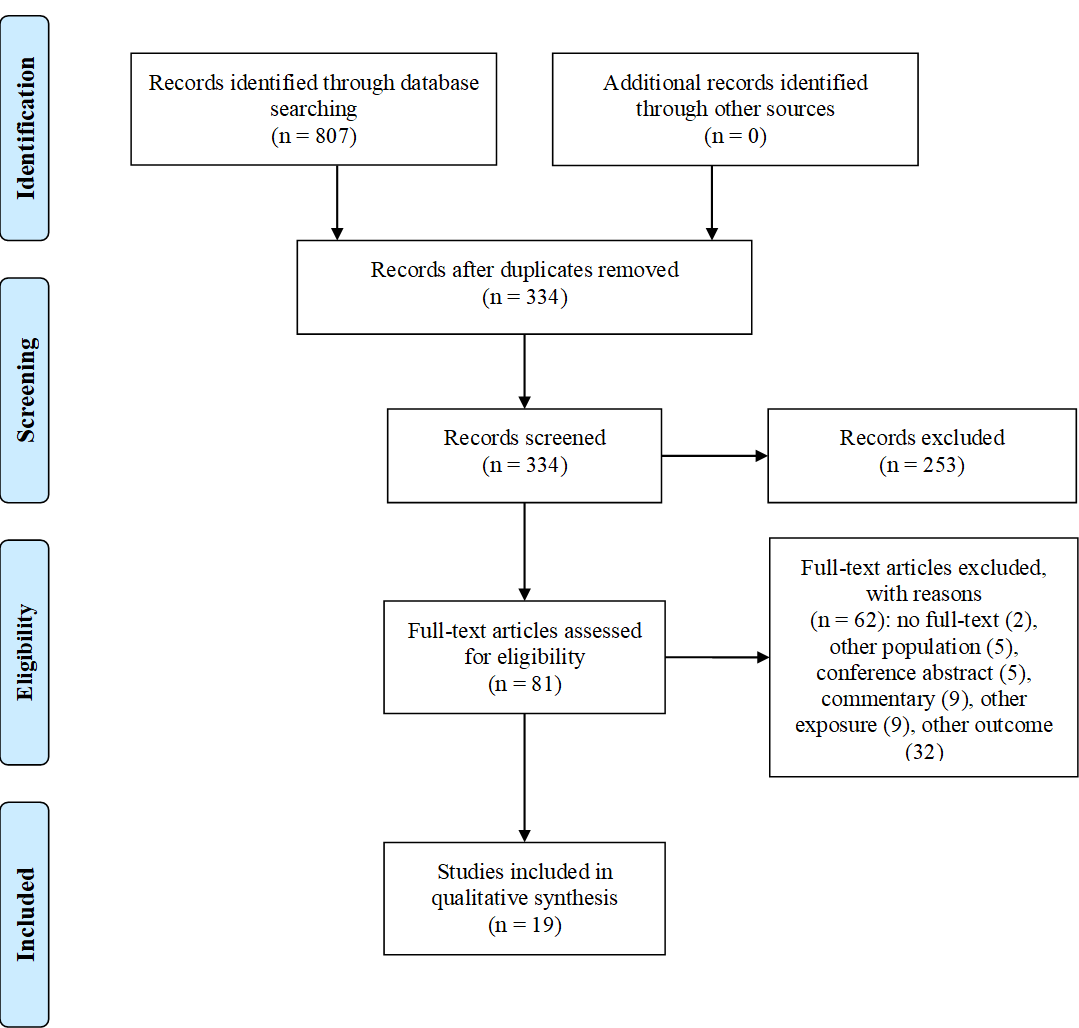


**Supplemental Figure 1. Flow diagram of study selection.**


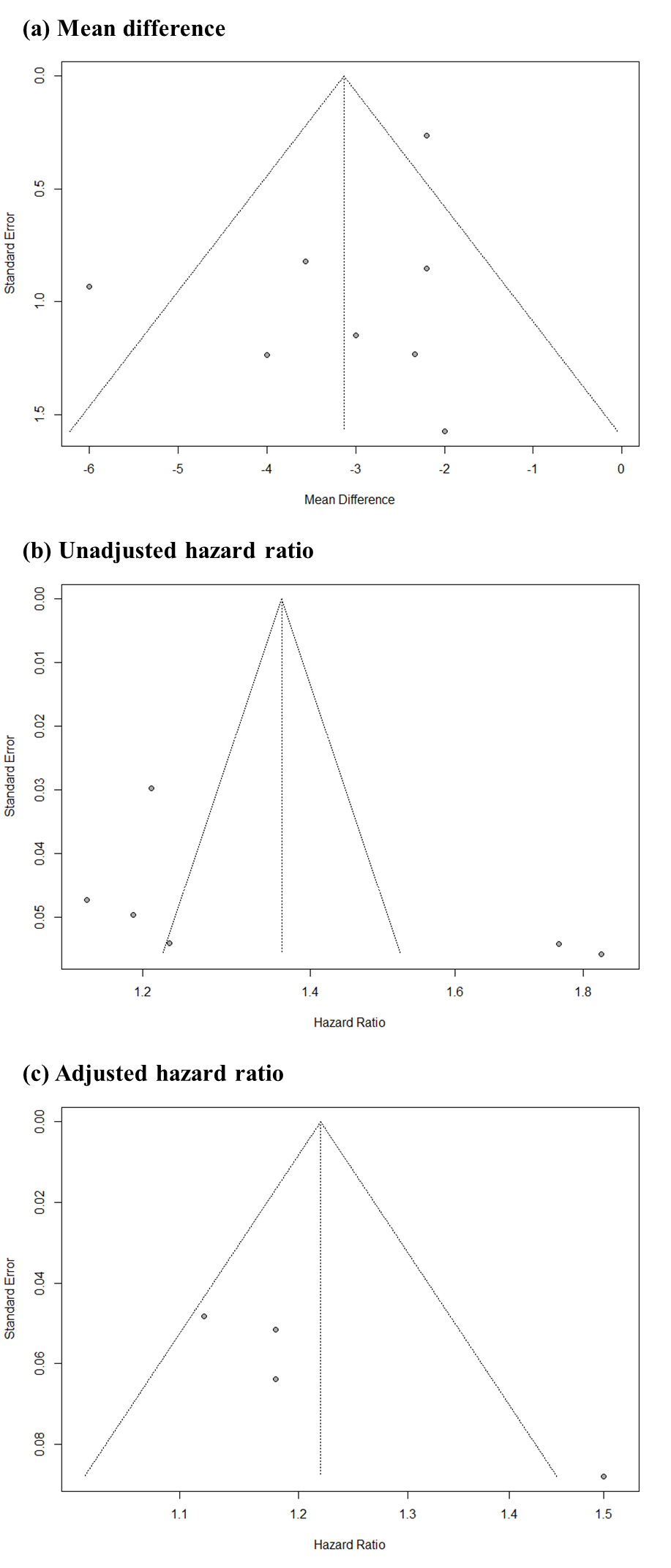


**Supplemental Figure 2. Funnel plots of tricuspid annular plane systolic excursion.**

**
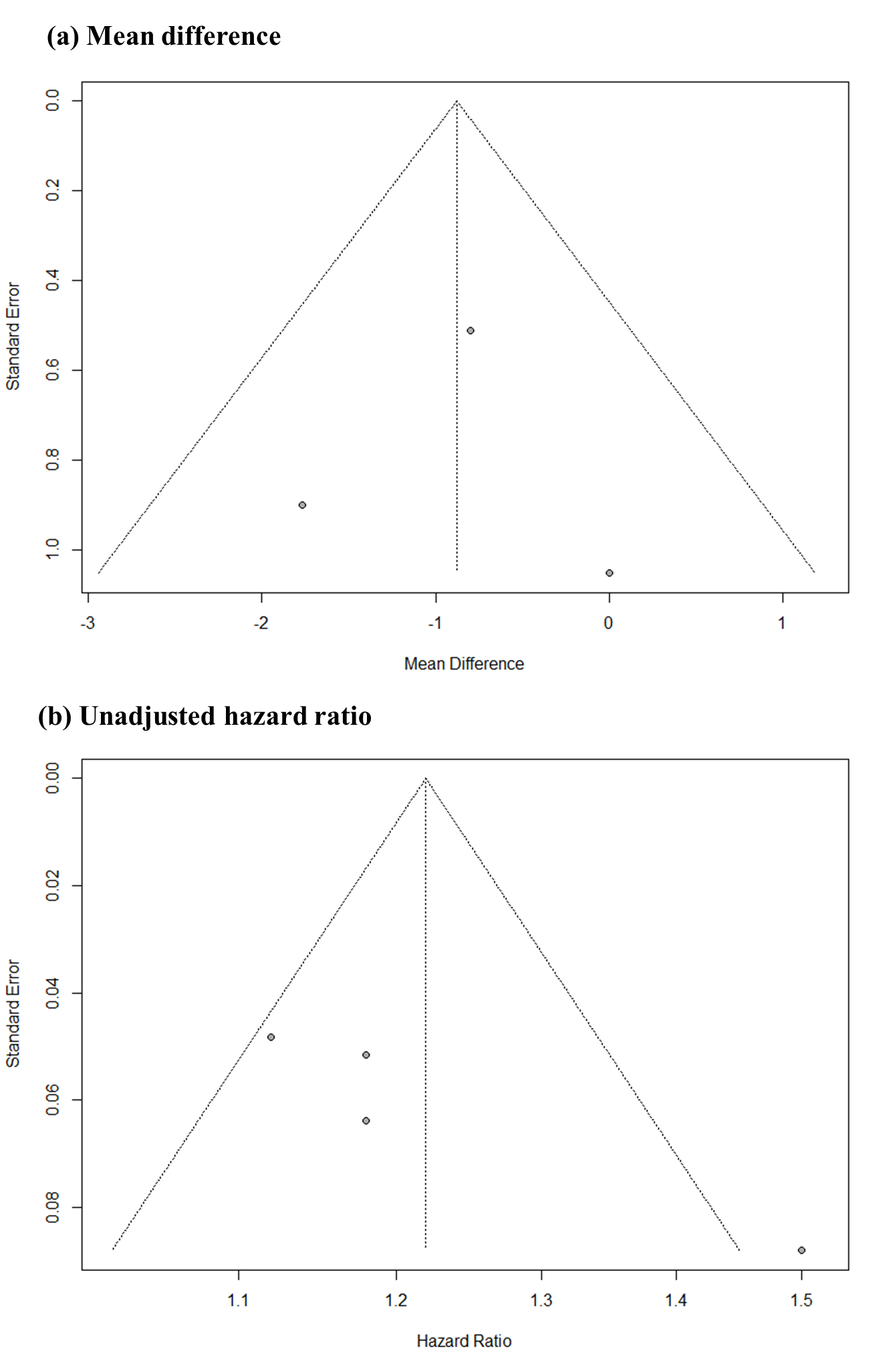
**

**Supplemental Figure 3. Funnel plots of tricuspid S’ systolic velocity.**


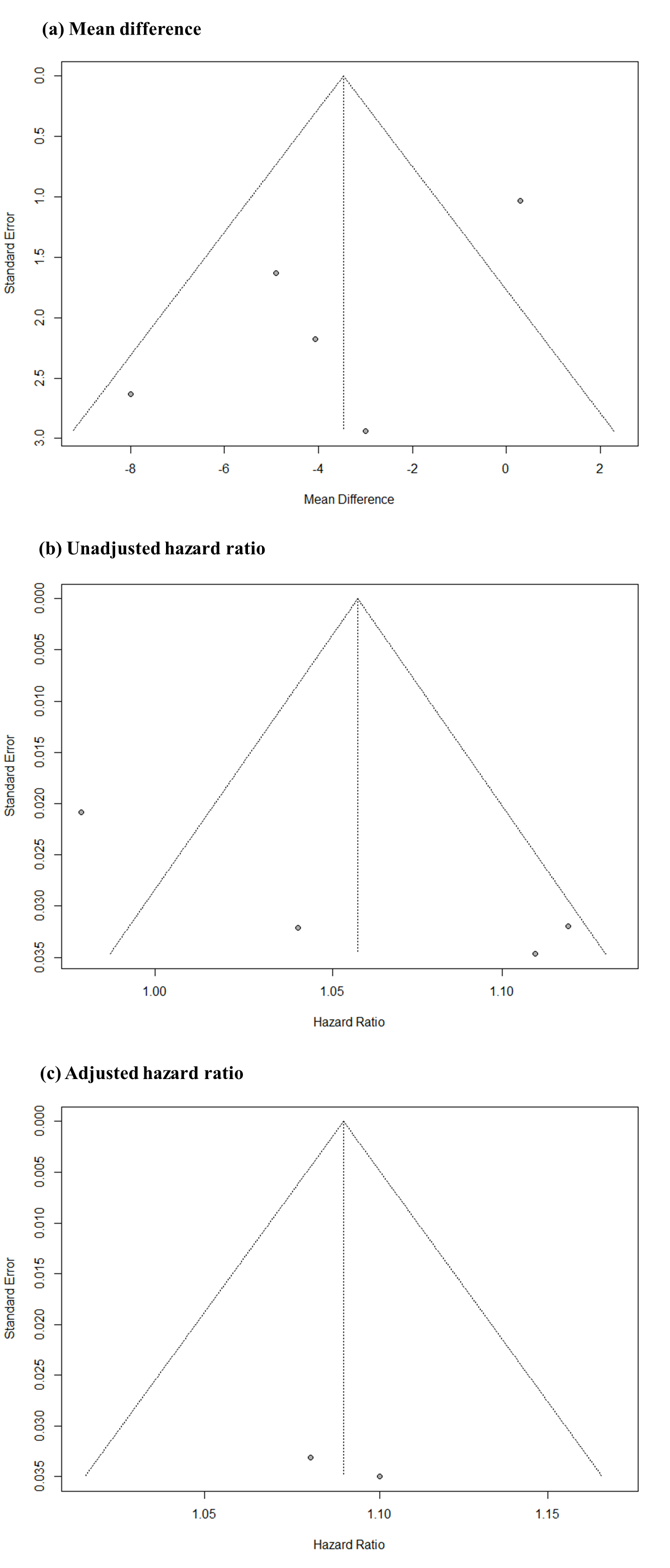


**Supplemental Figure 4. Funnel plots of fractional area change.**

**
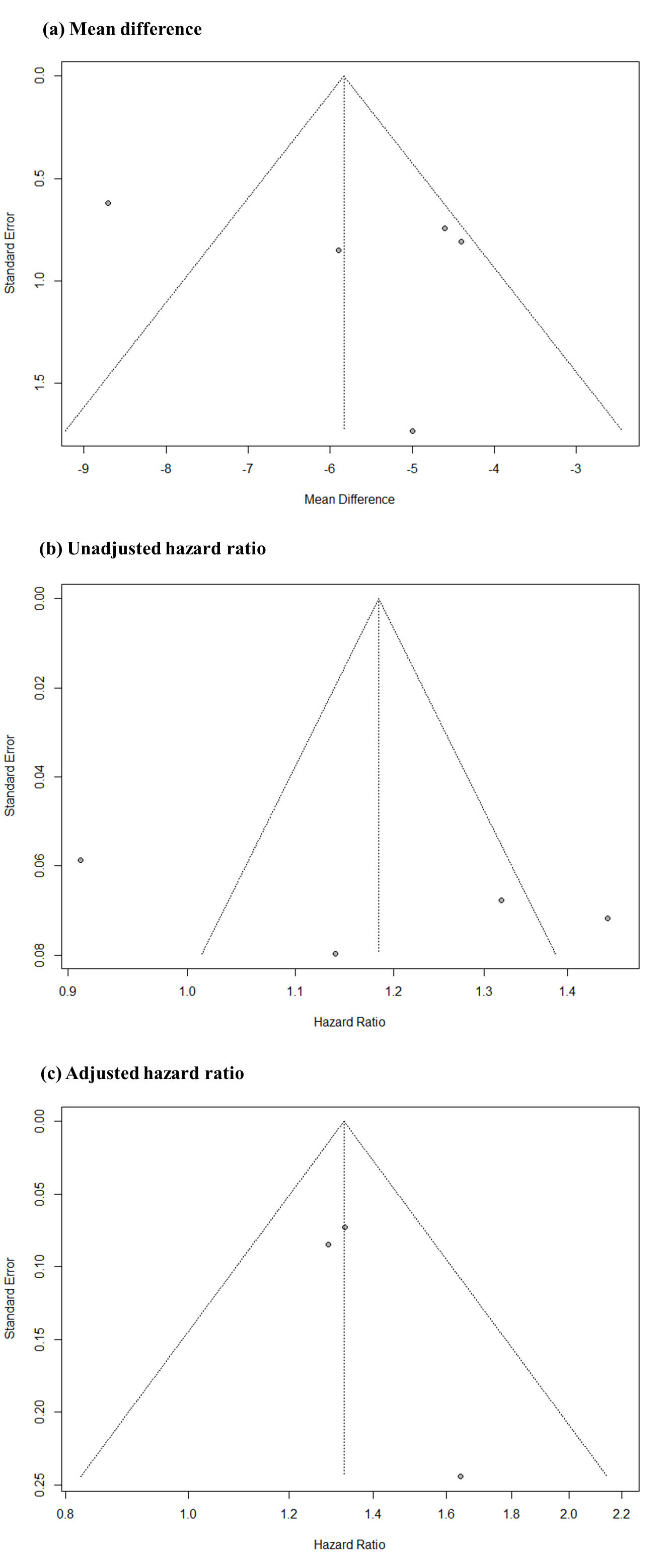
**

**Supplemental Figure 5. Funnel plots of right ventricular free wall longitudinal strain.**

**
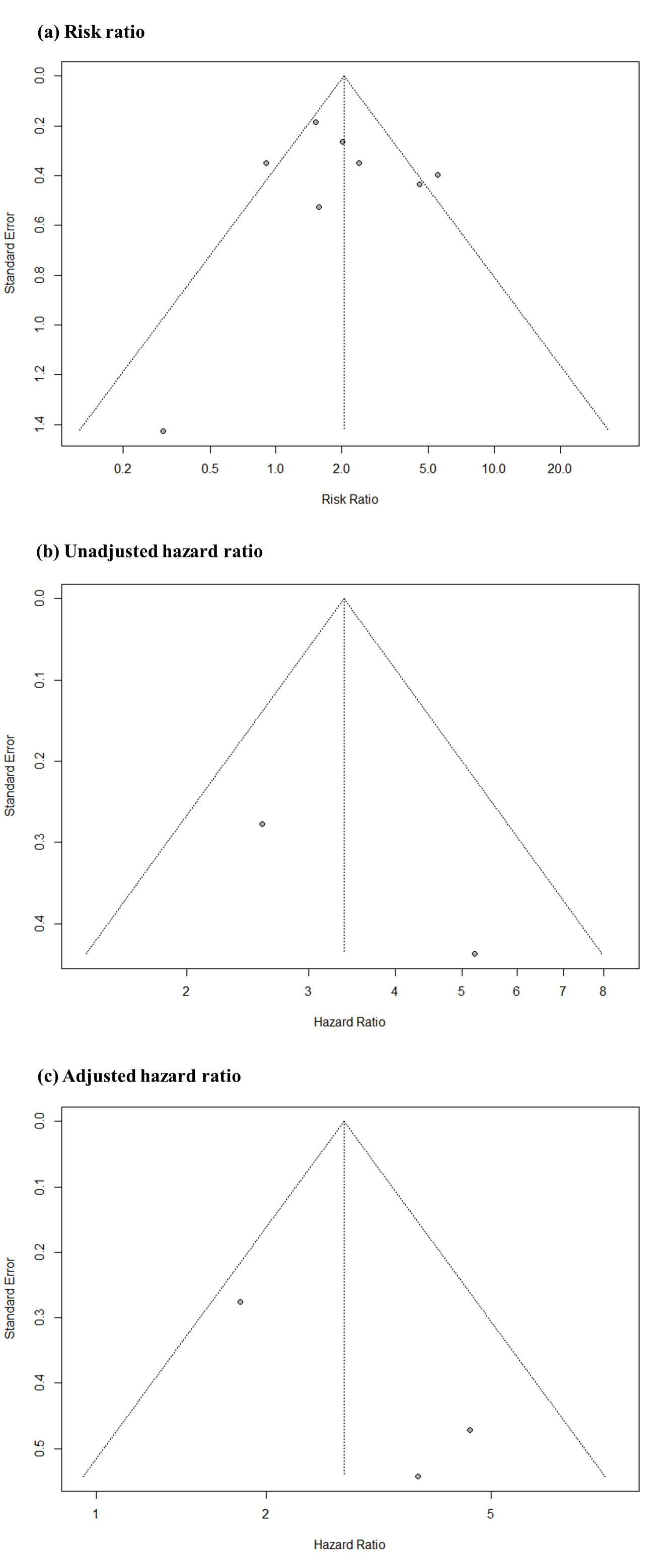
**

**Supplemental Figure 6. Funnel plots of right ventricular dysfunction.**

**
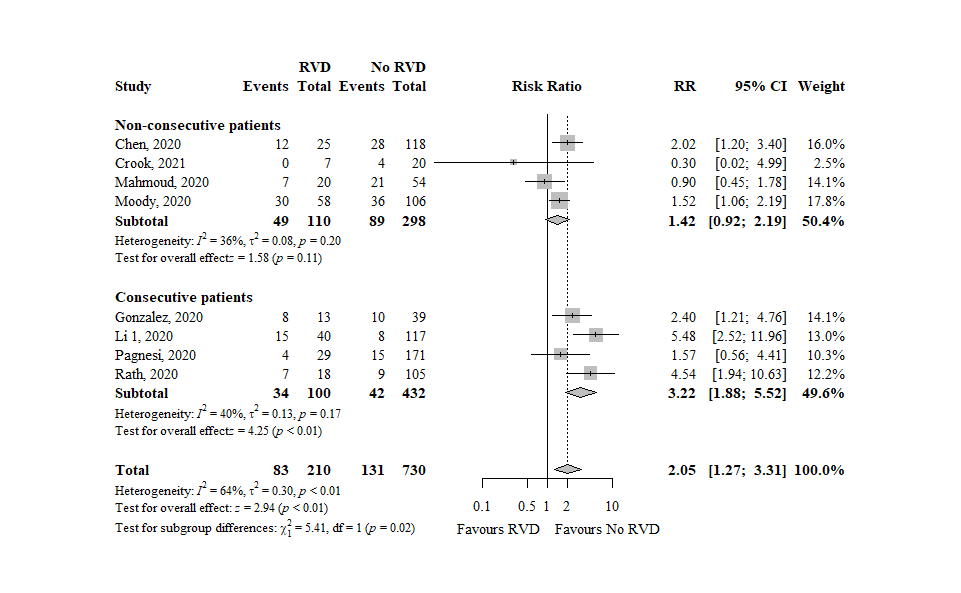
**

**Supplemental Figure 7. Forest plot showing the subgroup analyses according to type of population (consecutive versus non-consecutive). RVD, right ventricular dysfunction; RR, risk ratio; CI, confidence interval.**

**
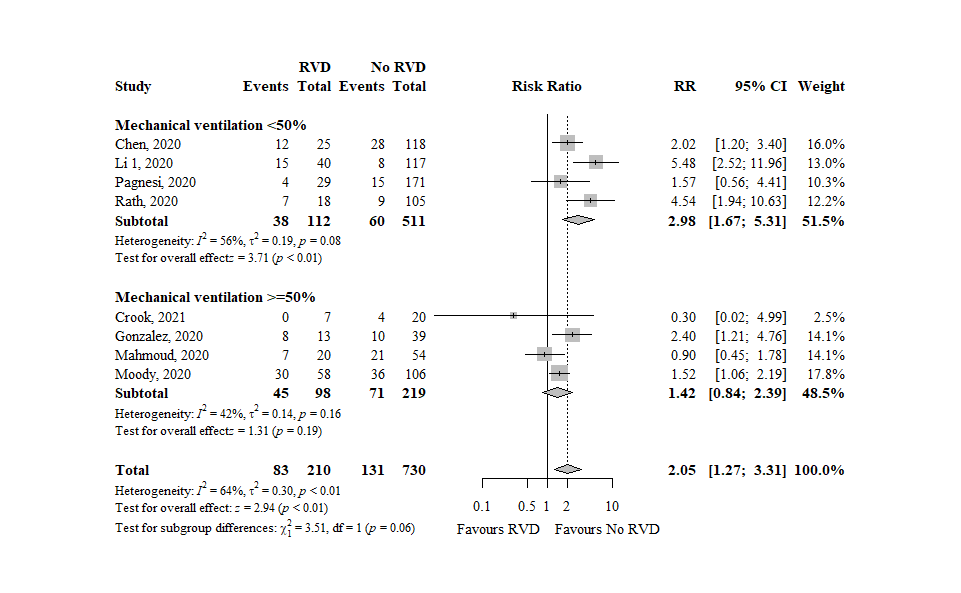
**

**Supplemental Figure 8. Forest plot showing the subgroup analyses according to proportion of mechanically ventilated patients (<50% versus ≥50%). RVD, right ventricular dysfunction; RR, risk ratio; CI, confidence interval.**
